# Books Review

**Published:** 1869-08

**Authors:** 


### Books Review.

*Soelberg Wells on Diseases of the Eye.* Philadelphia, HENRY C. LEA, 1869.

It is universally conceded that no department of medicine has changed so wholly, or rather grown so rapidly, as Ophthalmology. The discovery of the ophthalmoscope, and the development of the theories of refraction and accommodation, together with the changes which have taken place in the views of Ophthalmic Surgeons, as to the nature, causes and treatment of many of the diseases of the eye, have completely revolutionized the views of the profession, and constituted Ophthalmology a new science. This new work, by Soelberg Wells, embraces the modern doctrines of Ophthalmologists, and comprises the whole science as understood and practiced at the present day. It enters fully into all the advances which have lately been made, and embodies a complete record of the opinions and practices of the best authors. It contains two hundred and sixteen engravings on wood, which are very finely executed, and add greatly to a correct understanding of the text. There are, also, six colored plates, representing the ophthalmoscopic appearances of the normal *fundus occuli,* and the retina, as affected by various diseases. These colored plates are very fine, and are useful, indeed indispensable to the student who would understand the nature and causes of the diseased conditions of the retina as seen by aid of the Ophthalmoscope.

Speaking of Von Græfe's method of "Extraction of Cataract," which still receives a great deal of attention among Ophthalmologists, our author says: "The success of this operation has been so great that most Ophthalmologists, amongst whom I may mention Mr. Bowman, have abandoned the scoop extraction, and even to a great extent the flap operation. My own experience of it is also exceedingly favorable, and I prefer it greatly to every other operation." The subject of cataract is discussed at considerable length, and all the main questions at issue considered. This completeness is observed throughout the entire work; and this instance is only referred to, since our attention was directed to it as a subject upon which we would like to know the opinions of the author. The work appears to us as most admirably adapted to the wants of the profession, and we hope it will be in possession of all practitioners of medicine. Physicians, in general practice, are not expected to devote enough attention to the eye to become experts in the diagnosis or treatment of many of its more obscure or important diseases; but certainly all physicians should study the subject enough to understand how wholly incapable they are of giving advice in many cases which may involve the loss of vision. Every ophthalmic surgeon is often pained to witness total toss of vision from preventable causes, and in some instances, alas! from the want of appreciation of the danger by the family physician. Physicians, generally, should either bestow more attention to diseases of the eye, or refuse to give advice in all important cases; but physicians in the smaller towns cannot avoid the responsibility and should avail themselves of the advantages offered by such a work as the one before us. Similar works have been published within the last few years, but we venture to say that no one will be found to more fully meet the requirements of students and practitioners than the one by Soelberg Wells.

---

*Notes on the Phenols from Coal Tar, (Carbolic Acid) and on Rhubarb.* BY E. R. SQUIBBS, M. D.

These articles, which appeared in the Report of the Am. Pharm. Assoc., and in the Am. Jour. Pharm., are also presented us by the author in pamplet form. The paper on "Carbolic Acid" contains the results of recent original investigation, has also notes from general sources, and is of much interest to physicians as well as pharmacists. It is becoming generally understood that the carbolic acid of the shops contains two distinct products, phenol and cresol, together with more or less oily impurities. Pure crystallized carbolic acid is called phenol. The same name is also typical of a class of similar compounds. Cresol distils at higher temperatures than phenol, and is separated from it by fractional distillation.

The experiments on the comparative properties of these two substances result thus: "The azymotic power of their saturated solutions is somewhere about equal, whilst the saturated solution of phenol holds (6.6 per cent.) five times as much of that substance as a saturated solution of the other does of cresol (1.3 per cent.) Experiments, not yet complete, do not thus far show any marked difference in the antiseptic effect of the two phenols. In the writer's judgment there is no doubt as to the impropriety of separating and rejecting the cresol. A liquid mixture of the two phenols, in the ordinary proportions in which they occur, is at least equally useful for all the known purposes to which the crystallized carbolic acid has been applied in medicine or hygiene, whilst such mixtures are far more easily and cheaply obtained. It should not contain more than ten per cent. of insoluble impurities, and saturated solutions will contain from two to five per cent. of it, according as phenol or cresol predominate, but generally 4 per cent., while the standard saturated solution of crystalized phenol contains 5 per cent."

---

*Virchow's Life of the Trichina:* Translated by RUFUS KING BROWNE, M. D., New York.

The original German edition of this treatise of forty-eight pages, was published by Virchow, one of the early investigators of the subject, as being the only method of answering numerous individulal inquiries, and of enlightening the public, whom this matter so nearly concerns, to whom scientific works are not generally accessible. Dr. Browne has alike favored the English speaking community by his translation of the work.

---

*Proceedings of the Michigan State Medical Society for* 1867–8.

The report of the convention which organized the society in 1866, together with its proceedings for the two succeeding years are here given. Papers on Obstetrics, Diseases of Women, Zymotic Diseases, Lachrymal Obstructions, Surgery, etc., ten in number, are published, together with the annual address for 1868, by J. H. Jerome, M. D., of Saginaw City.

---

*American Exchange and Review.* FOWLER & MOON, Phila.

This monthly magazine, which has been published for the past seven years, represents a large variety of departments, economic and scientific, each of which receive due share of attention. Prof. H. S. Osborn, of Lafayette College, has charge of the mining and metallurgical department, and the esteem in which the publication is held by intelligent business corporations is well evinced by the patronage it receives.

---

*Transactions of the American Ophthalmological Society: Fourth and Fifth Annual Meetings.*

This volume was published, ready for distribution, at the last meeting of the society, held at Newport, in July. Twenty-five papers, which had been presented the society at the two previous meetings, are here printed, accompanied by test-letters and illustrative plates. The following amendment to their constitution, which is characteristic of the spirit of the Association, was passed last year:

"No member of this Society shall attach to his name, in any public announcement, the title of oculist, or any similar title, or shall announce in print that he gives special or exclusive attention to special practice."

*Transactions of the Georgia Medical Association.*

A revised Constitution and By-Laws were adopted by this Society last year. The annual meeting this year was held in May, when an address was delivered by L. A. Dugas, M. D., and reports made on Charlatanry, Medical Education, and the Axillary Thermometer. The code of ethics of the Am. Med. Assoc. is also published in the transactions which have been distributed to the physicians throughout the State.

---

*Our Home Physician: (Advance Sheets.)* By GEO. M. BEARD, A.M., M.D., Lecturer on Nervous Diseases in the University of New York, etc.

We most heartily sympathize with the effort to popularize science to a reasonable extent. We are confident that truth will vindicate itself among the people, in medicine just as surely as in astronomy; and those who are skilled themselves are to be the teachers of those who are not. The author of this work is a learned gentleman, and is assisted by some of the most eminent men of the profession, viz: Profs. D. B. St. John Roosa, Benj. Howard and others. As to the sound excellence of the work, we wish to call the attention of our readers to one of its articles, which we have re-printed in our present number.

---

*Good Health: A Journal of Physical and Mental culture.* Published monthly by ALEX. MOORE, 21 Franklin Street, Boston.

This periodical, the first number of which was issued in June, is a hygienic journal, in which subjects of health, recreation, intelligence and education are together treated of, so as to give the work popular interest. The effort to make the magazine instructive and entertaining has been successful. We are particularly interested in the articles on "The Eye and Sight," which are being contributed by B. Joy Jeffries, Ophthalmic Surgeon to Mass. Charitable Eye and Ear Infirmary. They present the latest established views on the subject, expressed very clearly, and made perfectly intelligible by means of correct diagrams. In an article of the present month, by the same writer, in the Boston Med. and Surg. Journal, he reviews the inaccuracies and inexcusable reticence of the text-books on Optics, which are used in our best colleges, and which profess to represent the present state of the science.

---

*Joined Twins—their Obstetrical and Surgical Management.* By A. B. COOK, A. M., M. D.

The case which suggested this monograph presents the following peculiarities, as summed up by the author: "1.—The junction of the zyphoid cartilages; 2.—Two linea albas; 3.—One common diaphragm; 4.—One common peritoneum, a lining, common cavity, and two sets of viscera with one exception; 5.—One common umbilical vein; 6.—One liver, with a double circulation; 7.—The curve of the inferior cava of the right fœtus to the left side of the vertebral column; 8.—The radical change in the relations of the vena cavas, hepatic veins and venous ducts to the posterior border of the liver; 9.—The duplication of all the organs except the liver."

*A Practical Manual of the Treatment of Club-Foot.* By Lewis A. Sayre, M. D. D. Appleton & Co., New York, 1869.

The author treats the subject in a systematic manner, describing in the first place the normal foot, complimenting it as being "one of the most beautiful examples of a complicated instrument, combining strength with mobility, that can be found in any part of the frame," He proceeds to discuss the causes of talipes, after having told what it is, and described its varieties. He says that the congenital forms are "all due to some interference, general or local, with the normal innovation of the part. So much has been generally accepted, but the real nature of this nervous disturbance has been for the most part misunderstood." The prevailing treatment by tenotomy he regards as based upon the theory of "spastic muscular contraction," and hence the therapeutical conclusion that tenotomy is a *sine qua non* of treatment. We have been greatly interested in the discussion of the causes of club-foot, partly because we have some theories and some facts of our own concerning it. Congenital club-foot is due, according to our author, to *some* interference with the normal innervation, but the cause of this innervation has been misunderstood. It has been supposed to be from spastic muscular contraction, but "the pathological change is precisely contrary to that which has been believed to exist. The muscles, supposed to be in a state of spasm, are really contracting with their normal degree of force, which produces an excessive effect, simply because paralysis of the opposing muscles has destroyed the natural harmony of action which exists between the tractile forces which govern the motions of the foot."

This theorising upon the causes of club-foot is as good as any—better than that by most authors. There is another plan of reasoning upon the subject, which appears to us as much more satisfactory; and when we write a monograph upon club-foot, and give our mode of dressing, we shall try to explain the whole philosophy of the thing. It appears remarkable that observers should wander off into the intangible, incomprehensible theories of enervation to explain the causes of club-foot, and other congenital deformities, when they can be demonstrated to arise from pressure during intra uterine life. We have several specimens which show this beyond all doubt. If pressure is such as to distort the form and prevent the motions and growth of the fœtus in utero, of course there is "some interference, general or *local*, with the normal innervation of the part." The authors modes of dressing are unexceptionable, and his results, as shown in the illustrative plates, are most satisfactory. We are most gratified to observe the stress laid upon the immediate treatment of congenital deformity, and regret that space does not permit us to notice this book more in detail.

---

*Quarterly Summary of the Transactions of the College of Physicians of Philadelphia.*

Nine interesting cases, which are here reported, have as subjects—Abortion, Bromide of Potassium in the sickness of Pregnancy, Drainage Probe, Fracture of the Skull of long standing, Fracture of base of Cranium, Acupressure of Femoral Artery, Paralysis, Epidemics and Meteorology. Memoirs are given of Drs. T. E. Beesley, C. W. Pennock and Francis West.

*The Intermarriage of Relations.* BY NATHAN ALLEN, M. D.

This article, which first appeared in the Psychological Quarterly, has received considerable attention from very many sources throughout the country. Dr. Allen is a thoroughly intelligent and active man, as his place on the board of Trustees of Amherst College would signify. He has interested himself greatly in the hygienic condition of our nation, and has lately published an article showing the condition of physical trainlng in the different colleges of our country, which reports quite in favor of a thorough system of gymnastic drill.

---

*The Probe.* By JOSEPH PARISH, M. D., Media, Pa.

This quarterly of thirty-two pages is issued from the "*Sanitarium*," an institution for persons who have become physically or mentally impaired through use of stimulants or narcotics, and which is under the charge of the editor, who devotes his journal to the discussion of the prevention and cure of such evils as are thus brought to his notice.

---

*Anniversary Oration delivered before the Medical Society of the District of Columbia by J. M. Toner, M. D., Sept. 26th,* 1866.

A historical review of the rise and growth of Medical science and its representatives in the District of Columbia, is here made celebrative of the forty-ninth anniversary of the society's birth. A period of two and a half centuries is embraced by the address, while a *resume* is given of the last century. The events given in chronological order are pleasantly narrated, and the statistical information quite abundant. A century ago there were but two physicians in Alexandria; its population in 1700 was 2749; that of Georgetown 1,200; while Washington, at the same time, was inhabited only by farmers and fishermen. At present the population of Washington is over a hundred thousand, and the number of physicians one hundred and fifty.

The following incident, which occurred in Washington during the prevalence of small pox in 1833, is narrated:—"President Jackson's coachman "Charles," a favorite servant, who had been with the General through all his Southern campaigns, was taken ill with the small pox. The case proved severe, and of a confluent form. The other servants about the *White House* were so much frightened, although immediately vaccinated, that it was impossible to get them to nurse him properly. When the General learned these facts, he did all he could to procure a competent nurse, but being unsuccessful he determined to assume these duties himself. He accordingly gave directions that he was not to be seen, and having changed his clothes he remained with "Charles" and gave him his medicine until he was considered ont of danger. I am indebted for the above incident to the General's family physician, *Dr. J. C. Hall.*

Epidemics, Hospitals, Contributors to Medical Literature, Medical Societies and Colleges, are particularly mentioned.

The society is to be congratulated on being able to publish so complete and entertaining a history.

